# Enhanced N-doped Porous Carbon Derived from KOH-Activated Waste Wool: A Promising Material for Selective Adsorption of CO_2_/CH_4_ and CH_4_/N_2_

**DOI:** 10.3390/nano9020266

**Published:** 2019-02-15

**Authors:** Yao Li, Ran Xu, Binbin Wang, Jianping Wei, Lanyun Wang, Mengqi Shen, Juan Yang

**Affiliations:** 1 School of Safety Science and Engineering, Henan Polytechnic University, Jiaozuo 454000, China; leayao35@aliyun.com (Y.L.); xuran950204@163.com (R.X.); lanyun.wang@gmail.com (L.W.); 13733838236@163.com (M.S.); 2 State Key Laboratory Cultivation Base for Gas Geology and Gas Control, Henan Polytechnic University, Jiaozuo 454000, China; 3 School of Materials Science and Engineering, Henan Polytechnic University, Jiaozuo 454000, China

**Keywords:** enhanced N-doped, porous carbon, absorbent, CO_2_/CH_4_ and CH_4_/N_2_, selectivity

## Abstract

Separation of impurities (CO_2_ and N_2_) from CH_4_ is an important issue for natural gas alternatives (such as coalbed gas, biogas, and landfill gas) upgrading. It is notably challenging to synthesize high N-doped porous carbon with an appropriate porous structure. In this work, high N content (14.48 wt %) porous carbon with micropore size of 0.52 and 1.2 nm and specific surface area of 862 m^2^ g^−1^ has been synthesized from potassium hydroxide (KOH) activated waste wool upon the urea modification. Pure component adsorption isotherms of CO_2_, CH_4_, and N_2_ are systematically measured on this enhanced N-doped porous carbon at 0 and 25 °C, up to 1 bar, to evaluate the gases adsorption capability, and correlated with the Langmuir model. These data are used to estimate the separation selectivities for binary mixtures of CO_2_/CH_4_ and CH_4_/N_2_ at different mixing ratios according to the ideal adsorbed solution theory (IAST) model. At an ambient condition of 25 °C and 1 bar, the predicted selectivities for equimolar CO_2_/CH_4_ and CH_4_/N_2_ are 3.19 and 7.62, respectively, and the adsorption capacities for CO_2_, CH_4_, and N_2_ are 2.91, 1.01, and 0.13 mmol g^−1^, respectively. This report introduces a simple pathway to obtain enhanced N-doped porous carbon with large adsorption capacities for gas separation of CO_2_/CH_4_ and CH_4_/N_2_.

## 1. Introduction

In latest decades, to satisfy the rapid growth in chemical and manufacturing industries, a large amount of fossil fuels burning has increased the level of carbon dioxide (CO_2_) in the atmosphere, which causes serious environmental issues, such as global climate change, the greenhouse effect, and ocean acidification [1,2,3]. A number of methods to reduce the CO_2_ gas have been suggested. One of the potential methods might be the use of natural gas (NG) as fuel for transportation and heating purpose [3]. Compared with the fossil fuels such as coal and petroleum, NG produces less CO_2_ per energy unit and has a high specific combustion enthalpy; therefore, it is considered a cleaner and potential substitute energy [4,5,6]. Additionally, NG has no threat to water or land, because of its non-toxic and no vapor pool forming on the ground as the liquefied petroleum gas does [3]. Nowadays, NG has provided a large amount of energy to satisfy business, industry, vehicle, and home needs throughout the world. The consumption of NG is expected to grow by 50% in the next 20 years, which makes the energy shortage situation much more serious [7]. As a result, coalbed gas, biogas, and landfill gas are deemed as attractive alternatives to NG, because they possess high methane (CH_4_) content and obvious socioeconomic advantages. However, the existence of some impurities such as CO_2_ and nitrogen (N_2_) will hamper the application of these alternative gases by reducing the heating value and corroding the transportation or storage system [7,8,9]. Consequently, urgent research is required to separate impurities (CO_2_ and N_2_) from CH_4_. At present, various technologies have been developed for gas separation and purification including cryogenic distillation, membrane separation, and pressure swing adsorption (PSA) [6,10]. Among these technologies, PSA has attracted intense interest due to its advantage of low cost, ease control, and energy effectiveness [6]. Accordingly, a lot of adsorbents have been evaluated for the adsorptive separation of CO_2_/CH_4_ and CH_4_/N_2_ such as metal–organic frameworks (MOFs) [11,12,13,14], organic zeolites [14,15,16], organic frameworks [17], and carbon-based materials [6,7,8,9,18,19].

Porous carbons have been considered as the most promising candidates for gas storage and separation because they are easy to synthesize and have good economical properties and strong thermal stability [20,21,22]. The adsorption of CO_2_, CH_4_, and N_2_ could be described as physisorption through weak van der Waals interaction forces [22]. Current research has focused on increasing the specific surface area (SSA) and controlling the pore size distribution (PSD), since the adsorption capacity is closely related to the textural characteristics of the carbon materials [22,23,24]. For example, Leventis et al. [25,26] reported that microporous carbon aerogels demonstrated an exceptionally high adsorption capacity for CO_2_. Meanwhile, for the purpose of improving the adsorption efficiency and further enhancing the interactions between gas molecules and carbon material, it is important to incorporate heteroatoms (i.e., N, P, S) into the carbon framework [21,22,25,26,27,28]. Recently, many efforts have been devoted to the synthesis of robust porous carbon that possesses high adsorption capacity and good selectivity, especially the N-doped porous carbon adsorbent. For example, the N-doped porous carbon synthesized from benzimidazole-linked polymers demonstrated high adsorption selectivity for CO_2_/CH_4_ and CO_2_/N_2_ [21]. The N-rich porous carbon through polyamine-incorporated metal–organic framework materials, which exhibited excellent CO_2_-selective adsorption ability over CH_4_ and N_2_ [27]. The N-doped graphene sheets by reducing graphene oxide sheets in the presence of polyindole displayed outstanding adsorption selectivity ratios for CO_2_ over N_2_, CH_4,_ and H_2_, respectively [28]. However, the synthesis processes of these N-doped porous carbons are very complex and time-consuming, and the precursor is not economical, which will enormously influence the practical application for the adsorbents. Thus, there still remains a challenge to develop porous carbon adsorbents with strong adsorption capacity, high selectivity, facile synthesis, and low cost.

Porous carbon adsorbents obtained from biomass materials are very competitive, which are not only cost-effective, easy to synthesis, abundant and sustainably renewable, but also happen to be excellent adsorbents for gas adsorption and separation [29,30,31,32]. So far, there already have been some biomass-derived porous carbons reported for CO_2_/CH_4_/N_2_ adsorption and separation. For example, Li et al. [31] investigated starch-derived porous carbon spheres for high uptakes of CO_2_, CH_4_, and H_2_. Niya et al. [32] synthesized N-doped carbon foams for CO_2_/N_2_, CO_2_/CH_4_, and CH_4_/N_2_ separation by using banana peel as a precursor and zinc complex as a template. Because of the N-rich nature, waste wool is a desirable precursor for the fabrication of N-doped porous carbon [33]. In a previous study, we have successfully synthesized N-doped hierarchical porous carbon from waste wool by potassium hydroxide (KOH) activation, exhibiting a good selective adsorption capability of CO_2_/N_2_ [34]. Although KOH activation is a well method for developing the porosity structure of the carbon, it unavoidably leads to a loss of N from the carbon framework [33,34,35].

**Figure 1:**
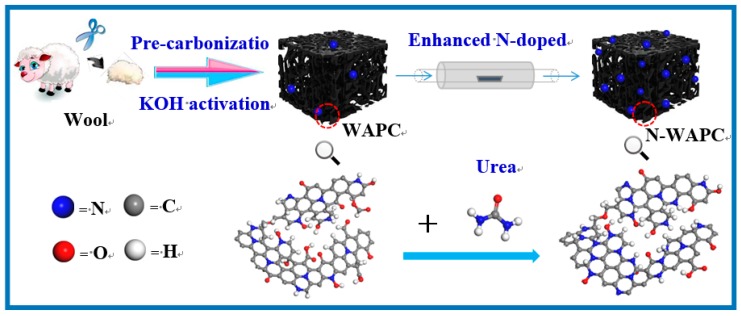
Schematic illustration of the synthesis of enhanced N-doped porous carbon.

Therefore, enhancing the N content of the porous carbon after KOH activation is an urgent task for further gas selective adsorption application. Keeping the above purpose in mind, in this work we propose a simple method to synthesize N-enhanced porous carbon from KOH activated waste wool for selective adsorption of CO_2_/CH_4_ and CH_4_/N_2_. As shown in Figure 1, first waste wool is chosen as carbon precursor to be activated by KOH, and then modified by urea. Here, waste wool is chosen because it is largely composed of keratin amino acids and is a unique renewable natural material with high N contents. However, the KOH activation can make a significant evolution of the structure for the original wool derived activated porous carbon (WAPC), which simultaneously reduces the N groups and introduces many O groups into the carbon frameworks [34,35,36]. Upon the urea modification, a significant amount of N groups can be again introduced into the activated carbon matrix, because the high N content (46 wt %) urea can react with O-containing functional groups [37]. The CO_2_, CH_4_, and N_2_ adsorption properties are evaluated at 0 °C and 25 °C, respectively, on the as-obtained enhanced N-doped wool derived activated porous carbon (N-WAPC) The isotherm data are used to predict the separation selectivities for binary mixtures of CO_2_/CH_4_ and CH_4_/N_2_ assuming different mixing ratios by the ideal adsorption solution theory (IAST).

## 2. Experimental Section

### 2.1. Materials

Waste wool was obtained from rural pastures of Henan, China. Potassium hydroxide (KOH), hydrochloric acid (HCl), and urea (NH_2_CONH_2_) were purchased from Sinopharm Chemical Reagent Co. Ltd. China (Beijing, China) and used as received without any further purification.

### 2.2. Sample Preparation

First, the waste wool was thoroughly washed, and then pre-carbonized at 300 °C under N_2_ atmosphere. The as-obtained black and crisp carbide was ground to powder and mixed with a KOH agent (the ratio of KOH/pre-carbonized sample = 3), activated under N_2_ atmosphere as we have previously reported [34]. The resulting waste wool derived activated porous carbon was denoted as WAPC. In the following synthesis, 0.1 g of WAPC and 0.1 g of urea were thoroughly mixed by grinding for 20 min. After that, the mixture was transferred into a quartz boat placing in a horizontal tube furnace. Subsequently, the mixture was calcinated at 600 °C for 1 h with a heating rate of 3 °C min^−1^ under N_2_ atmosphere. After cooling down, the sample was thoroughly washed with hot distilled water to remove the residual urea, and then dried at 60 °C to obtain the final product. The as-obtained N-enhanced porous carbon sample was denoted as N-WAPC.

### 2.3. Material Characterizations

Scanning electron microscopy (SEM) images and elemental mappings were obtained by field emission scanning electron microscopy (FE-SEM, Hitachi S-4800, Hitachi, Tokyo, Japan) with an energy dispersive X-ray spectrometer (EDS) (Hitachi, Tokyo, Japan). Transmission electron microscopy (TEM) and high-resolution TEM (HR-TEM) measurements were carried out on a JEOL-2100F transmission electron microscope (JEOL, Tokyo, Japan) operated at 200 KV. Powdered X-ray diffraction (XRD) patterns were performed on a Bruker-D8 powder diffractometer (Bruker, Madison, WI, USA) using Cu Ka radiation (λ = 0.15406 nm). Raman spectra were collected on a Renishaw inVia (Renishaw, London, UK) with an excitation wavelength of 514 nm. Fourier transform infrared (FT-IR) spectra were collected on a Nicolet iS10 FT-IR spectrophotometer (Nicolet, Madison, WI, USA), using the KBr pellet technique. X-ray photoelectron spectroscopy (XPS) was performed on a Thermo Scientific ESCALAB250 (Thermo Fisher Scientific, Waltham, MA, USA) equipped with an Al Ka excitation source. Elemental analysis (C, H, and N) was performed on a dry basis using a VarioEL III Elemental Analyzer (Elementar, Hanau, Germany). The N_2_ adsorption–desorption isotherms were measured on a Quantachrome NOVA1000e volumetrically sorption analyzer (Quantachrome, Boynton Beach, FL, USA) at liquid nitrogen temperature. Prior to each adsorption experiment, the degassing procedure was carried out for 3 h at 300 °C to remove the guest molecules in the sample. The SSA was calculated by multiple points Brunauer–Emmett–Teller (BET) method and PSD was determined by a non-local density functional (NLDFT) theory using nitrogen adsorption data and assuming a slit pore model.

### 2.4. Adsorption Measurements

The CO_2_, CH_4_, and N_2_ adsorption isotherms were measured by the Micromeritics ASAP 2020 volumetrically at two temperatures of 0 °C and 25 °C, respectively. Ultrahigh-purity CO_2_, CH_4_, and N_2_ were used as received. As aforementioned, the degassing procedure was also carried out for 3 h at 300 °C prior to the adsorption measurement.

## 3. Results and Discussions

### 3.1. Characterizations of the As-Prepared Samples

**Figure 2:**
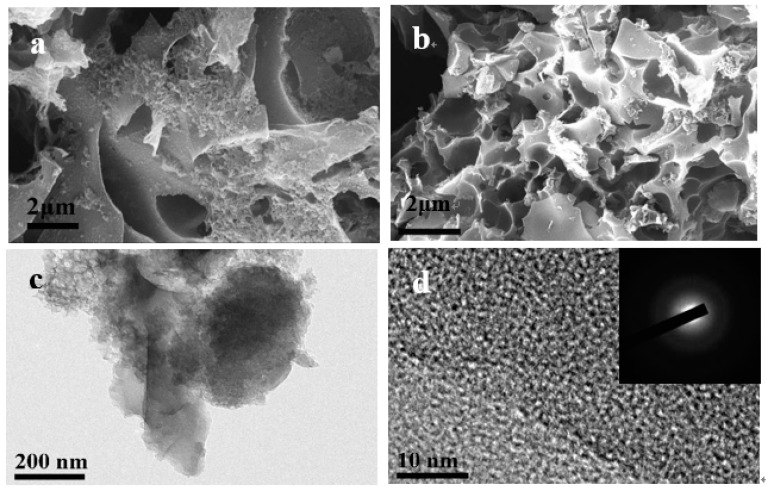
SEM images of (**a**) wool derived activated porous carbon (WAPC), (**b**) N-doped wool derived activated porous carbon (N-WAPC); TEM image (**c**,**d**) HRTEM image of N-WAPC (inset: SAED).

The SEM images of the pre-carbonized sample show a bulky morphology with a smooth surface, and no pores can be observed (Figure S1). After the KOH activation process, the activated sample WAPC exhibits an obviously developed porous structure with spongy pores on the surface (Figure 2a). Further urea nitridation, the structure of the N-enhanced sample N-WAPC becomes more or less collapsed; however, the whole sponge-like porous structure still can be well kept (Figure 2b). TEM and HR-TEM measurements are also carried out to study the overall morphology and microstructure of the N-WAPC. As showed in Figure 2c, a continuous sponge-like architecture with a connected porous texture is clearly observed. Furthermore, the HR-TEM image of N-WAPC (Figure 2d) shows an amorphous curved surface with worm-like micropores, as well as the selected area electron diffraction (SAED) pattern exhibiting typical diffuse rings (inset in Figure 2d).

**Figure 3:**
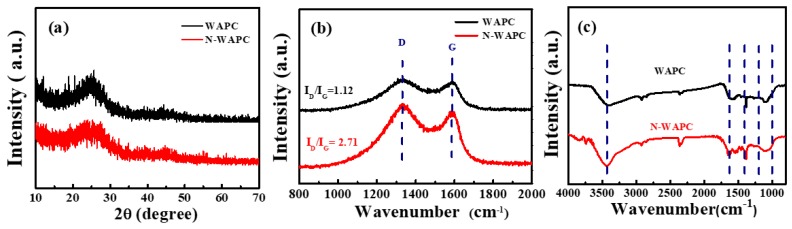
Powder XRD patterns (**a**), Raman spectra (**b**), and FT-IR spectra of WAPC and N-WAPC (**c**).

XRD and Raman are employed to characterize the phase structures of the porous carbon samples. As shown in Figure 3a, the two broad low-intensity diffraction peaks around 25° and 43° correspond to the (002) and (100) diffraction patterns of amorphous graphitic carbon [38,39,40]. The weak peaks suggest a low graphitization of the WAPC. After urea nitridation treatment, these two peaks become much weaker, indicating a more disordered graphitic structure of N-WAPC. In Figure 3b, the two bands centered at about 1340 cm^−1^ (D band) and 1590 cm^−1^ (G band) are separated completely. The D band is closely related to the presence of disordered carbon structures, whereas the G band is associated with the sp^2^-hybridized carbon atoms in a graphitic layer [38,39,40]. The intensity ratio of the D and G band (I_D_/I_G_) is known to be an indicator of the degree of graphitization, the higher the I_D_/I_G_, the lower the degree of graphitization [39]. In our case, the I_D_/I_G_ peak ratios for WAPC and N-WAPC are 1.12 and 2.71, respectively, indicating a low degree of graphitization. The I_D_/I_G_ ratio of N-WAPC is higher than that of WAPC, suggesting more defect sites are introduced in N-WAPC during the urea nitridation, which corresponds with the XRD result.

The FT-IR spectroscopy is used to investigate the chemical bonding information of the porous carbon samples (Figure 3c). The broad absorption band around 3430 cm^−1^ is attributed to the N–H and/or O–H stretching vibrations [41,42,43]. The band at about 1580 cm^−1^ represents N–H in-plane deformation vibration or C=C stretching vibration in aromatic rings [42,43]. The characteristic absorption at 1418 cm^−1^ corresponded to C–N stretching vibration [44]. The broad band around 1200–1000 cm^−1^ can be assigned to the C–N or C–O stretching vibration [41,42]. The spectra of WAPC and N-WAPC present similar broad and overlapping absorption bands, which can be attributed to the strong absorption of carbon [45]. Furthermore, the N-WAPC exhibits some relatively strong amplitude, which associates with nitrogen-related bands of N–H and C–N.

The elemental analysis is used to provide chemical compositions of the carbon samples, and the corresponding results are demonstrated in Table 1. The N contents are found to be 4.14 and 14.48 wt % for the WAPC and N-WAPC, respectively, while the O contents are 24.79 and 12.44 wt %, and the C contents are 69.65 and 71.98 wt %. Clearly, the WAPC has a significant high O content, which should be ascribed to the KOH activation treatment [34]. The high level of oxygen-containing functional groups in the WAPC makes it very reactive for subsequent urea nitridation [37]. After the urea treatment, the N contents increase from 4.14 wt % for WAPC to 14.48 wt % for N-WAPC. On the contrary, the O contents decrease from 24.79 wt % for WAPC to 12.44 wt % for N-WAPC. While the C contents for the two samples do not change significantly. Above results demonstrate that the oxygen-containing groups on the WAPC can interact with the amino groups in the urea, contributing to the incorporation of N into the carbon framework.

**Figure 4:**
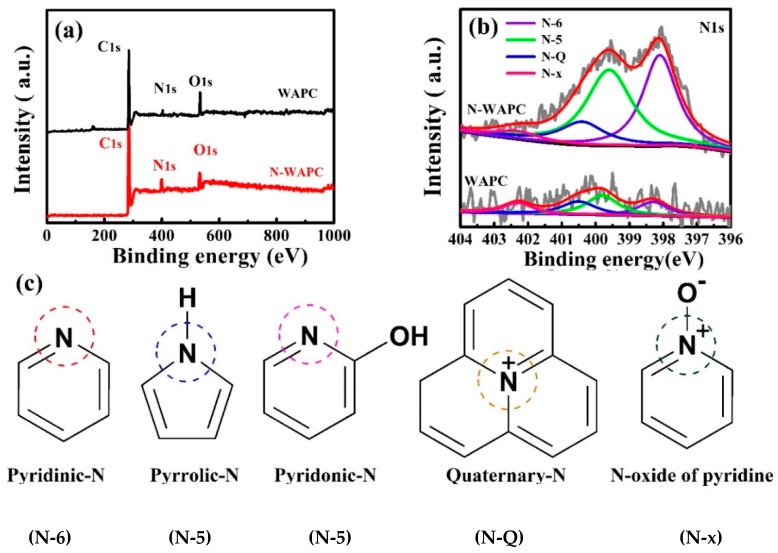
XPS for WAPC and N-WAPC (**a**) full-survey spectra and (**b**) high-resolution spectra of N1s; (**c**) the different N species possibly present in the porous carbons.

In order to further investigate the feature of surface states, the XPS technique is applied for the carbon samples. As shown in Figure 4a, both samples exhibit C1s, N1s, and O1s peaks, respectively. It is noteworthy that the N-WAPC has a considerably strong N1s peak compared with WAPC (Figure 4a). The corresponding N species are presented in Figure 4b. The peaks at ~398.1 eV, ~400.0 eV, ~400.7 eV, and ~402.8 eV are attributed to pyridinic-N (N-6), pyrrolic-/pyridonic-N (N-5), quaternary-N (N-Q), and N-oxides of pyridine (N-x), respectively [43,44,46,47,48]. The structure of these different N species is shown in Figure 4c. To the best of our knowledge, the pyrrolic-N and pyridonic-N cannot be well distinguished from each other within the accuracy of XPS measurements. However, considering the oxygen presenting in the porous carbons and the fact that pyridonic-N is more stable than pyrrolic-N, the pyridonic-N is more likely to be present in the porous carbons [42]. As to pyridonic-N, the p orbital in its –OH would produce p–π conjugation effect with its p bond, which endows the N located in the ortho-position of –OH with stronger electron atmosphere and consequently enhances its Lewis basicity, resulting in the improvement of the CO_2_ adsorption ability of porous carbons [42]. Thus, the pyridonic-N, acting as an anchor for CO_2_ capture, is beneficial for CO_2_ capture [42,43]. Whereas, other N species have no active effect for CO_2_ capture [46]. Quantitative analysis calculations from the N1s peak area show that the N amounts of form N-5 is higher than those present of form N-6, N-Q and N-x, in the order of N-5 > N-6 > N-Q > N-x for both samples of WAPC and N-WAPC (Table 1). It should be noted that after urea nitridation treatment, the percentage of form N-5 in N-WAPC increases significantly. The relative percentage of N-5 in WAPC is only 33%, while in N-WAPC it is 44% (Table 1). This would be favorable for CO_2_ capture, because N-5 contributes more significantly to capturing CO_2_ than other forms of nitrogen species [46]. The high-resolution spectra of C1s and O1s for WAPC and N-WAPC are shown in Figure S2. The C1s peak can be deconvoluted to six sub-peaks at 284.5, 285.3, 285.9, 286.7, 287.3, and 288.8 eV, respectively (Figure S2a). The main peak centers at 284.5 eV corresponds to C-C [49], while the other weak peaks can be identified as C-OH at 285.3 eV [36,49], C=N at 285.9 eV [49], C-O-C at 286.4 eV [48], N-C=O at 287.4 eV [49], and -COOH at 288.8 eV [48]. Three characteristic peaks are observed at 531.8, 532.5, and 533.4 eV in the O 1s spectra (Figure S2b), which can be ascribed to C=O, C-OH, and O-C-O, respectively [48,49,50]. Furthermore, from the SEM energy dispersive X-ray spectroscopy (EDS) element mapping, it can be seen that N, C, and O elements are uniformly distributed on the N-WAPC surface (Figure S3).

**Figure 5:**
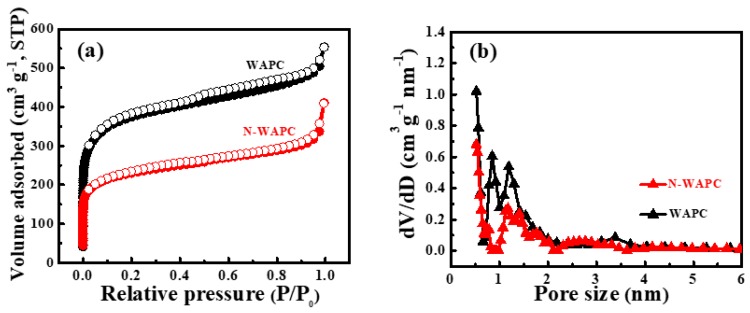
(**a**) N_2_ adsorption–desorption isotherms and (**b**) pore size distribution (PSD) curves of WAPC and N-WAPC.

N_2_ adsorption–desorption isotherms of WAPC and N-WAPC and the corresponding PSD curves are shown in Figure 5. The isotherms of WAPC and N-WAPC (Figure 5a) display a combination form of type I and type IV, with a rapid N_2_ uptake at very low relative pressure (P/P_0_) and a slight hysteresis loop at medium P/P_0_ range, indicating the presence of rich micropores and a small number of mesopores in the samples [51]. However, the N_2_ uptake amount at low P/P_0_ region for N-WAPC is obviously less than that of WAPC, which can be attributed to the structural collapse that resulted from urea nitridation. Correspondingly, the PSD curves of WAPC and N-WAPC calculated by the NLDFT theory both exhibit the presence of micropores and mesopores (Figure 5b). The PSD curve for WAPC displays three relatively strong micropore peaks at about 0.52, 0.85, and 1.2 nm, respectively, and one weak mesopore peak around 3.4 nm. As for N-WAPC, the two micropore peaks mainly center at around 0.52 and 1.2 nm, and one very weak mesopore peak at about 2.6 nm. As can be seen clearly, the peaks intensities of micropores and mesopores for N-WAPC are much inferior to that of N-WAPC, which is in accordance with their N_2_ adsorption isotherms and microstructure results. As shown in Table 2, which includes BET SSA (S_BET_) that calculated from N_2_ adsorption branch and total pore volume (V_tot_) that obtained from N_2_ adsorption amount at P/P_0_ = 0.99, respectively. The S_BET_ for WAPC and N-WAPC is 1352 and 862 m^2^ g^−1^, respectively; and the V_tot_ for WAPC and N-WAPC is 0.74 and 0.5 m^3^ g^−1^, respectively. We note that both the S_BET_ and the V_tot_ significantly decrease after nitridation treatment with urea, because the structure collapse and addition N-groups may block partial pore volume.

### 3.2. Gas Adsorption and Separation Studies

**Figure 6:**
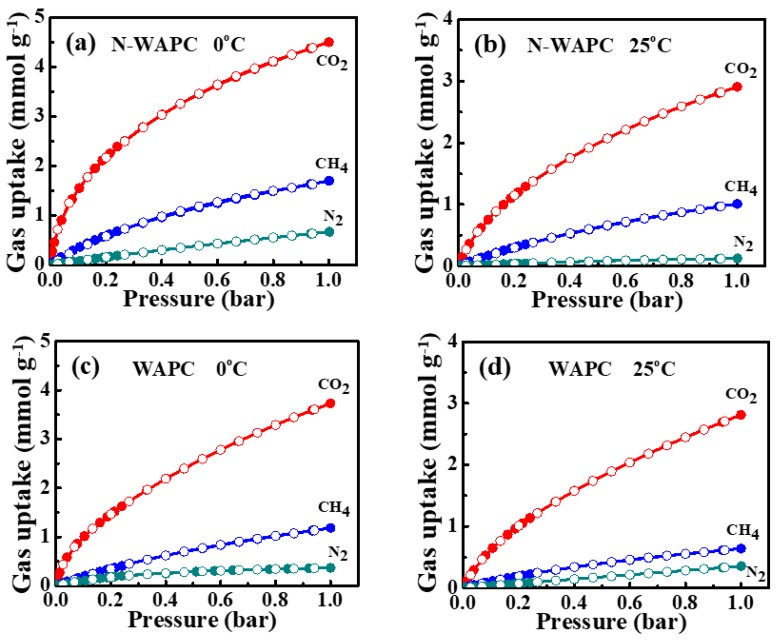
Gas uptake isotherms of CO_2_, CH_4_, and N_2_ for (**a**) N-WAPC at 0 °C, (**b**) N-WAPC at 25°C, (**c**) WAPC at 0 °C (**d**) WAPC at 25 °C.

The pure component adsorption isotherms of CO_2_, CH_4_, and N_2_ for WAPC and N-WAPC are measured at two temperatures (25 °C and 0 °C) and pressure up to 1 bar, as shown in Figure 6 and summarized in Table 2. All the isotherms show excellent reversibility without hysteresis, suggesting that the adsorbed molecules can be well removed during the desorption process. Thus, the samples of WAPC and N-WAPC can be easily regenerated by a vacuum without any heating treatment, which makes these samples superior to some MOFs and zeolites materials [6]. The adsorption isotherms for CO_2_ are fitted by the Double Site Langmuir (DSL) model and the adsorption isotherms for CH_4_ and N_2_ are fitted by the Langmuir (L) model. The calculation details and parameters are summarized in Figure S4–S9 and Table S1–S3 in the supporting information. It is clear that the amounts of gases adsorbed varied widely for samples N-WAPC and WAPC, in the sequence of CO_2_ > CH_4_ > N_2_. In both WAPC and N-WAPC, CO_2_ is the most strongly adsorbed gas owing to its significant quadrupolar moment and polarizability [6,9,52]. CH_4_ shows stronger adsorption than N_2_, which is attributed to its higher polarizability than that of N_2_ [6,9,52].

The adsorption capacity is an important factor to assess the gas separation capability for an adsorbent [6]. The higher CO_2_ capacity is obtained by N-WAPC, which is measured to be 4.50 and 2.91 mmol g^−1^ at 0 and 25 °C, respectively. These values are superior to some recently reported carbon-based adsorbents: sOMC (2.0 mmol g^−1^), MAC (2.13 mmol g^−1^), and N-HPCMs-5-0.6-973 (1.82 mmol g^−1^) at 25 °C and 100 kPa [6,7,53]. Although the S_BET_ and V_tot_ for the WAPC are high, the CO_2_ capacity for WAPC (only 3.73 and 2.81 mmol g^−1^ at 0 and 25 °C, respectively) is much lower than that of N-WAPC. This can be clarified by the significantly enhanced N content in N-WAPC (14.48 wt %), especially the high percentage of form N-5 in N-WAPC (44%). Furthermore, the N-WAPC also exhibits high CH_4_ adsorption capacities of 1.70 and 1.01 mmol g^−1^ at 0 and 25 °C, respectively, at the pressure of 1 bar. As for the N_2_ uptake, the N-WAPC shows adsorption capacities of 0.67 and 0.13 mmol g^−1^ at 0 and 25 °C, respectively, under the pressure of 1 bar. Under the same conditions, all these values outperform the WAPC adsorption capacities on CH_4_ (1.18 mmol g^−1^ at 0 °C and 0.64 mmol g^−1^ at 25 °C) and N_2_ (0.37 mmol g^−1^ at 0 °C and 0.35 mmol g^−1^ at 25 °C). 

In addition to the desirable gas uptake, selectivity is also very important for potential application in gas separation. Considering the good gas adsorption capacity of N-WAPC, the gas separation capability of N-WAPC for CO_2_/CH_4_ and CH_4_/N_2_ are further measured. The IAST is a widely used method to predict the binary mixture gas adsorption selectivity behavior based upon single component gas adsorption results [6,9,34]. Here, we use IAST to predict the selectivity of N-WAPC for the binary mixtures (CO_2_/CH_4_ and CH_4_/N_2_). In a mixture, the selectivity of the preferential adsorption gas 1 over gas 2 can be formally defined as the following equation:

S_ads_ = [q_1_/q_2_]/[p_1_/p_2_] (1)

where S_ads_ is the selectivity factor, q_1_ and q_2_ are the absolute adsorbed loadings at a partial pressure of p_1_ and p_2_, respectively, in the binary mixture. The IAST selectivities of the binary mixtures at different gas proportions on N-WAPC in this paper are typical components of CH_4_-related gases, such as coalbed gas, biogas, and landfill gas. Accordingly, the mixing ratios of 50/50, 15/85, and 5/95 for CO_2_/CH_4_, and CH_4_/N_2_ are used to calculate the selectivity and the overall performance is summarized in Table 3.

**Figure 7:**
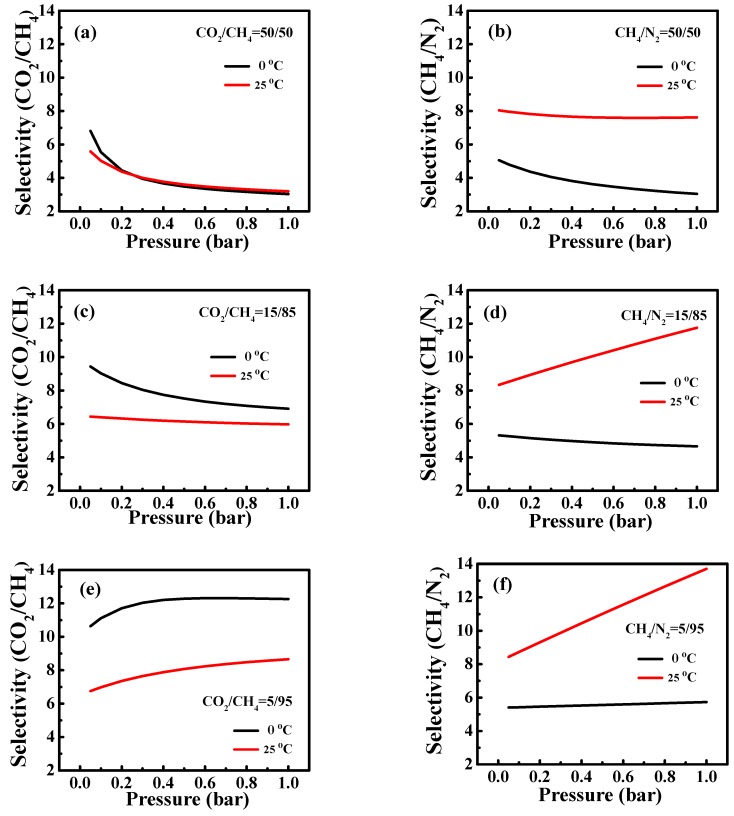
IAST-predicted selectivities assuming different binary mixing ratios on N-WAPC (**a**) CO_2_/CH_4_ = 50/50, (**b**) CH_4_/N_2_ = 50/50, (**c**) CO_2_/CH_4_ = 15/85, (**d**) CH_4_/N_2_ = 15/85, (**e**) CO_2_/CH_4_ = 5/95, and (**f**) CH_4_/N_2_ = 5/95 at 0 and 25 °C.

The selectivities of binary mixtures for CO_2_/CH_4_ and CH_4_/N_2_ on N-WAPC, are plotted as a function of total bulk pressure in Figure 7. For equimolar binary mixture (50/50) of CO_2_/CH_4_, the selectivities decrease with the increased pressure, obtaining about 3.03 (0 °C) and 3.19 (25 °C) at 1 bar (Figure 7a). The CH_4_/CO_2_ selectivity displayed by N-WAPC is comparable to or higher than some other reported adsorbents [6,54,55,56]. When it comes to the CH_4_/N_2_ (50/50) separation, the selectivities are also decreased with the increased pressure and obtained 3.04 (0 °C) and 7.62 (25 °C) at 1 bar (Figure 7b), which is comparable to that of ultra-microporous carbon [57] and polycarbazole polymers under the same condition [58]. The selectivities for the binary mixture CO_2_/CH_4_ and CH_4_/N_2_ at other mixing ratios are further predicted by IAST. As shown in Figure 7c, with the mixture ratio of 15/85 for CO_2_/CH_4_, although the selectivities are decreased with the increased pressure, it can maintain a relatively high level of 6.91 (0 °C) and 5.98 (25 °C) at 1 bar. Whereas in the mixture ratio for CO_2_/CH_4_ is 5/95, the selectivity situation is different. As shown in Figure 7e, the CO_2_/CH_4_ selectivities gradually increase with the increased pressure, finally reaching 12.26 (0 °C) and 8.66 (25 °C) at 1 bar. As for the binary mixture of CH_4_/N_2_, when the gas component is 15/85, the CH_4_/N_2_ selectivity slightly decreases with the increased pressure, keeping 4.66 at 0 °C and 1 bar (Figure 7d). However, at 25 °C, the CH_4_/N_2_ selectivity obviously increases with the increased pressure, reaching about 11.76 under the pressure of 1 bar (Figure 7d). For the case of binary mixture CH_4_/N_2_ with mixing ratio of 5/95, the selectivities increase with the increased pressure, reaching 5.73 (25 °C) and 13.71 (0 °C) at 1 bar (Figure 7f). It is worth noting that, in the same conditions, the selectivities of CO_2_/CH_4_ and CH_4_/N_2_ keep increasing in the composition range (50/50 ~ 15/85 ~ 5/95), which is an attractive feature of an adsorbent. The listed selectivities indicate that the as-synthesized N-WAPC is a promising adsorbent in gas adsorptive separation.

**Figure 8:**
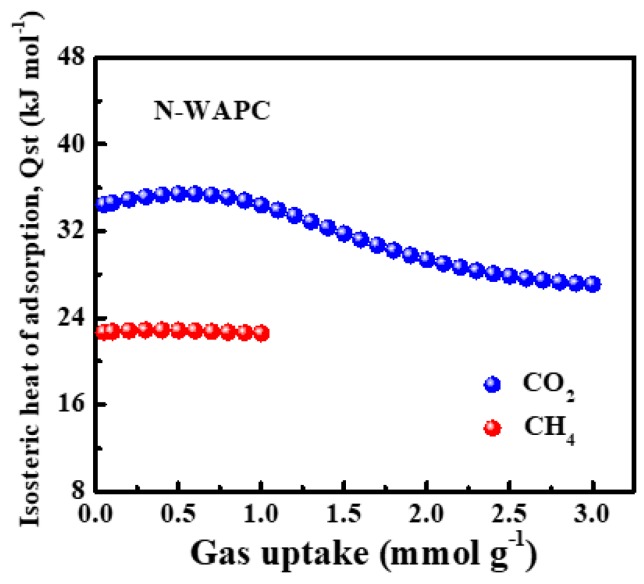
Isosteric heats of adsorption for CO_2_ and CH_4_ on the N-WAPC.

The isosteric heat of adsorption (Q_st_) is the standard enthalpy of adsorption at a fixed surface coverage [59], which is an important value to evaluate the interactions between gas molecules and adsorbents and further elucidates the selectivity. The Q_st_ for CO_2_ and CH_4_ on N-WAPC are calculated by obtaining the CO_2_ and CH_4_ isotherms at two different temperatures (25 and 0 °C). The single component Q_st_ as a function of surface loading can be determined by a variant of the Clausius–Clapeyron equation as following [59,60,61]:

(2) $$\ln\left(P_{1}{/P}_{2}\right)\;=\;\Delta H_{\mathrm{ads}}\;(\frac{T_{2}\;{-\;T}_{1}}{{R\;T}_{1\;}T_{2}})$$

where ΔH_ads_ gives the isosteric heat of adsorption. P_1_ and P_2_ are the pressures, for the same amount of gas uptake, at two different temperatures T_1_ and T_2_ respectively. R is the ideal gas constant. As can be seen clearly in Figure 8, the Q_st_ values of CO_2_ is in the range of 35.44–27.10 kJ mol^−1^ gently dropping from low coverage to saturation, which means that the interaction between CO_2_ molecules and the absorbent is stronger than that between CO_2_ molecules [58]. The Q_st_ of CH_4_ is almost in the horizontal range from 22.59 to 22.88 kJ mol^−1^, which apparently exceeds standard enthalpy for interaction of CH_4_ with carbon (16 kJmol^−1^) and many activated carbon materials, such as M1273-150 (17–11 kJ mol^−1^) [31], C1000 (17.4–16.3 kJ mol^−1^) [62], K-PAF-1 (20.6–15.9 kJ mol^−1^) [63]. The enhanced N-doped porous carbon with appropriate pore structure can greatly enhance CH_4_ adsorption through weak interactions, and can therefore achieve a high CH_4_/N_2_ selectivity. Furthermore, the Q_st_ of CO_2_ is obviously higher than that of CH_4_ within the experiment range, indicating a high CO_2_/CH_4_ selectivity. These high selectivities demonstrate that the enhanced N-doped porous carbon has potential in applications for CH_4_-related gas upgrading, such as coalbed gas, biogas, and landfill gas.

## 4. Conclusions

Enhanced N-doped porous carbon with an appropriate pore structure has been synthesized from KOH activated waste wool upon the urea modification. The as-obtained porous carbon N-WAPC has a high N content of 14.48 wt %, SSA of 862 m^2^ g^−1^, and an appropriate micropore size of 0.52 and 1.2 nm. The gas the adsorption capacities of N-WAPC follows the order CO_2_ > CH_4_ > N_2_. The adsorption selectivities of binary mixtures CO_2_/CH_4_ and CH_4_/N_2_ were predicted by the IAST method. At 25 and 1 bar, the selectivities of N-WAPC for equimolar CO_2_/CH_4_ and CH_4_/N_2_ are 3.19 and 7.62, respectively. The good selective adsorption performance of N-WAPC reveals its great potential for efficient methane mixture gases upgrading.

## Figures and Tables

**Table 1:** Elemental composition and relative percentages of nitrogen species of the carbon samples.

Sample	Elemental Composition (wt %)				Relative Percentages of Nitrogen Species ^[b]^ (%)			
	C	N	H	O ^[a]^	N-6	N-5	N-Q	N-x
WAPC	69.65	4.14	1.42	24.79	25	33	25	17
N-WAPC	71.98	14.48	1.10	12.44	40	44	12	4

^[a]^ Calculated by difference. ^[b]^ Calculated from the corresponding peak areas of the deconvoluted N1s spectra.

**Table 2:** Textural characteristics and gas uptakes for WAPC and N-WAPC.

Samples	Textural Characteristics		Gas Uptakes ^[c]^ (mmol g^−1^)					
	S_BET_^[a]^ (m^2^g^−1^)	V_tot_^[b]^ (cm^3^g^−1^)	CO_2_		CH_4_		N_2_	
			0 °C	25 °C	0 °C	25 °C	0 °C	25 °C
WAPC	1352	0.74	3.73	2.81	1.18	0.64	0.37	0.35
N-WAPC	862	0.50	4.50	2.91	1.70	1.01	0.67	0.13

^[a]^ S_BET_ is the specific surface area calculated by BET equations based on the adsorption data in the P/P_0_ range from 0.005 to 0.05. ^[b]^ V_tot_ is the total pore volume obtained at P/P_0_ ~ 0.99. ^[c]^ Gas uptake in mmol g^−1^ is obtained under 1 bar.

**Table 3:** The selectivity of N-WAPC for binary mixtures ^[a]^.

Temp.	CO_2_/CH_4_			CH_4_/N_2_		
	50/50	15/85	5/95	50/50	15/85	5/95
0 °C	3.03	6.91	12.26	3.04	4.66	5.73
25 °C	3.19	5.98	8.66	7.62	11.76	13.71

^[a]^ Calculated from IAST( 0 and 25 °C) for the binary mixtures assuming different mixing ratios at 1 bar.

## Supplementary Materials

The following are available online at http://www.mdpi.com/2079-4991/9/2/266/s1, Figure S1: SEM image of the pre-carbonized waste wool; Figure S2: High-resolution XPS spectra of (a) C1s and (b) O1s for WAPC and N-WAPC; Figure S3: (a) SEM image for N-WAPC and the corresponding EDS element mappings of (b) N, (c) C and (d) O; Figure S4: CO_2_ gas adsorption for N-WAPC at 0 °C (a) and 25 °C (b). The continuous solid line corresponds to the DSL fittings of the experimental data; Figure S5: CH_4_ gas adsorption for N-WAPC at 0 °C (a) and 25 °C (b). The continuous solid line corresponds to the L fittings of the experimental data; Figure S6: N_2_ gas adsorption for N-WAPC at 0 °C (a) and 25 °C (b). The continuous solid line corresponds to the L fittings of the experimental data; Figure S7: CO_2_ gas adsorption for WAPC at 0 °C (a) and 25 °C (b). The continuous solid line corresponds to the DSL fittings of the experimental data; Figure S8: CH_4_ gas adsorption for WAPC at 0 °C (a) and 25 °C (b). The continuous solid line corresponds to the L fittings of the experimental data; Figure S9: N_2_ gas adsorption for WAPC at 0 °C (a) and 25 °C (b). The continuous solid line corresponds to the L fittings of the experimental data; Table S1: Langmuir parameters and coefficient of determination for adsorption of CO_2_ in N-WAPC and WAPC; Table S2: Langmuir parameters and coefficient of determination for adsorption of CH_4_ in N-WAPC and WAPC; Table S3: Langmuir parameters and coefficient of determination for adsorption of N_2_ in N-WAPC and WAPC.
