# Supplementary material for: Enhanced N-doped Porous Carbon Derived from KOH-Activated Waste Wool: A Promising Material for Selective Adsorption of CO2/CH4 and CH4/N2
Source: Nanomaterials (Basel). 2019 Feb 15;9(2):266. doi: 10.3390/nano9020266 (PMC6409997; doi:10.3390/nano9020266)
Supplement: Supplementary file 1 [file nanomaterials-09-00266-s001.pdf]

Supplementary information

Enhanced N-doped porous carbon derived from KOH activated waste wool: a promising material for selective adsorption of CO_2/CH_4 and CH_4/N_2

Yao Li [1,2,†], Ran Xu [1,†], Binbin Wang [3,*], Jianping Wei [1,2,*], Lanyun Wang [1], Mengqi Shen [1] and Juan Yang [1,*]

[1] School of Safety Science and Engineering, Henan Polytechnic University, Henan 454000, China; leayao35@aliyun.com (Y.L.); xuran950204@163.com (R.X.); lanyun.wang@gmail.com (L.W.); 13733838236@163.com (M.S.)

[2] State Key Laboratory Cultivation Base for Gas Geology and Gas Control, Henan Polytechnic University, Henan 454000, China.

[3] School of Materials Science and Engineering, Henan Polytechnic University, Henan 454000, China.

[†] These authors contributed equally

* Corresponding authors: wangbb@hpu.edu.cn (B.W); weijianping@hpu.edu.cn (J.W.); yangjuan@hpu.edu.cn (J.Y.); Tel.: +86-391-398-6901 (B.W.); +86-391-398-7885 (J.W.); +86-391-398-7881 (J.Y.)

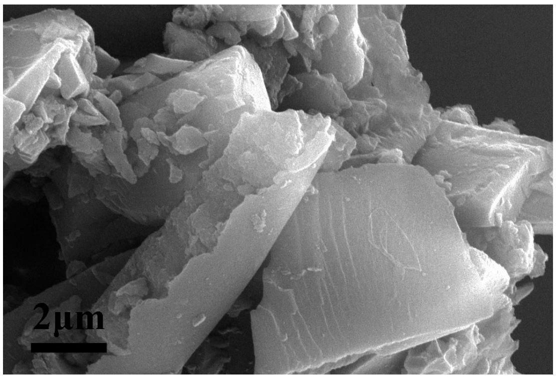

Figure S1. SEM image of the pre-carbonized waste wool.

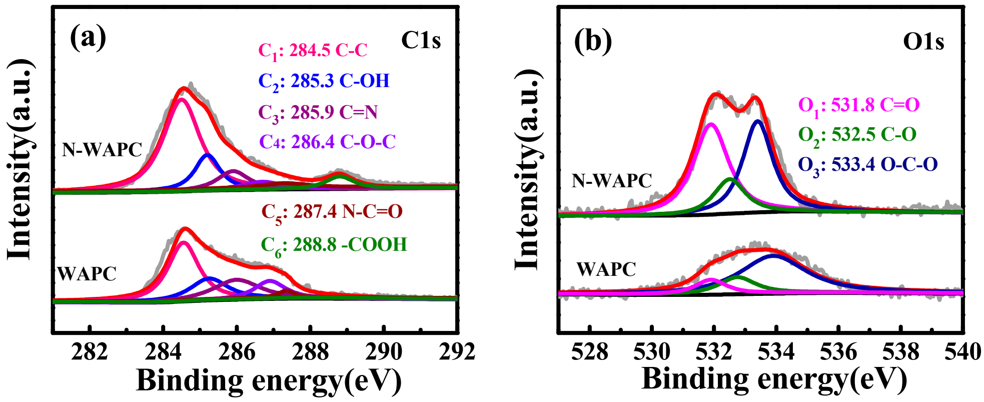

Figure S2. High-resolution XPS spectra of (a) C1s and (b) O1s for WAPC and N-WAPC.

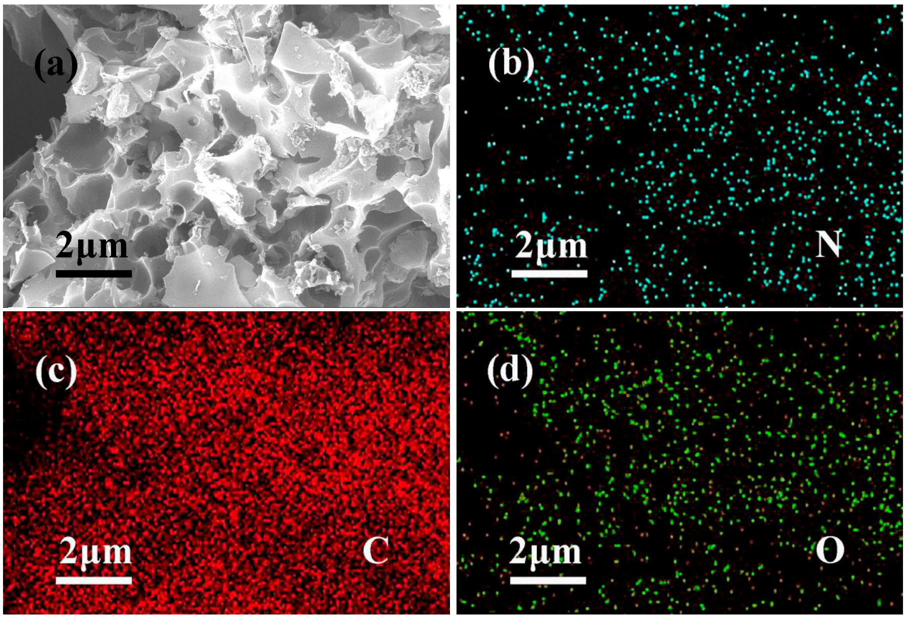

Figure S3. (a) SEM image for N-WAPC and the corresponding EDS element mappings of (b) N, (c) C and (d) O.

Fitting of pure component isotherms

The experimentally measured uptakes for CO_2, CH_4, and N_2 are measured as a function of the absolute pressure at two different temperatures of 25 ºC and 0 ºC. The isotherm data for CO_2 in N-WAPC and WAPC are fitted with the Double Site Langmuir (DSL) model, as the isotherm data for CH_4 and N_2 are fitted with the Langmuir (L) model.

L model:

$$q = \frac{q_{sat,B}\, bp}{1 + bp}$$

DSL model:

$$q = q_A + q_B = \frac{q_{sat,A}\, b_A p}{1 + b_A p} + \frac{q_{sat,B}\, b_B p}{1 + b_B p}$$

Where q is the amount of gas adsorbed (mmol/g), p is the pressure (bar), q_{sat} is the saturation capacity (mmol/g), b is the Langmuir parameter (bar^{-1}). For CO_2 isotherms the DSL model ($q = q_A + q_B$) is employed to get a reasonable fitting. The A and B are two distinct adsorption sites.

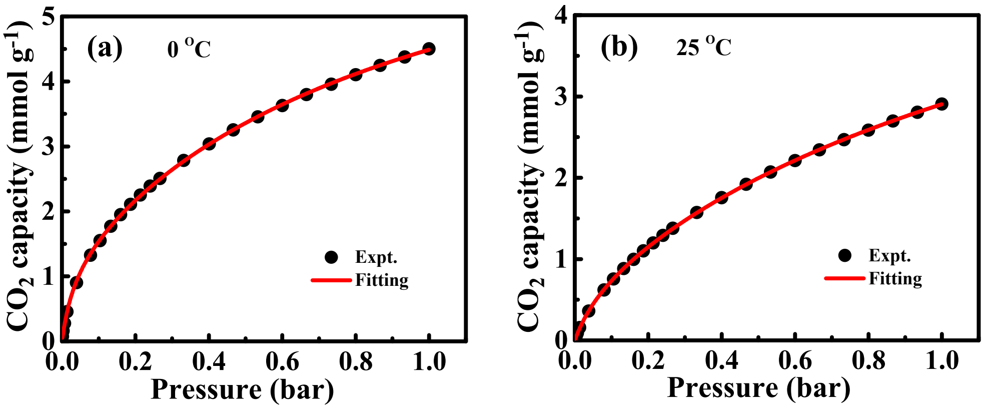

Figure S4. CO_2 gas adsorption for N-WAPC at 0 ºC (a) and 25 ºC (b). The continuous solid line corresponds to the DSL fittings of the experimental data.

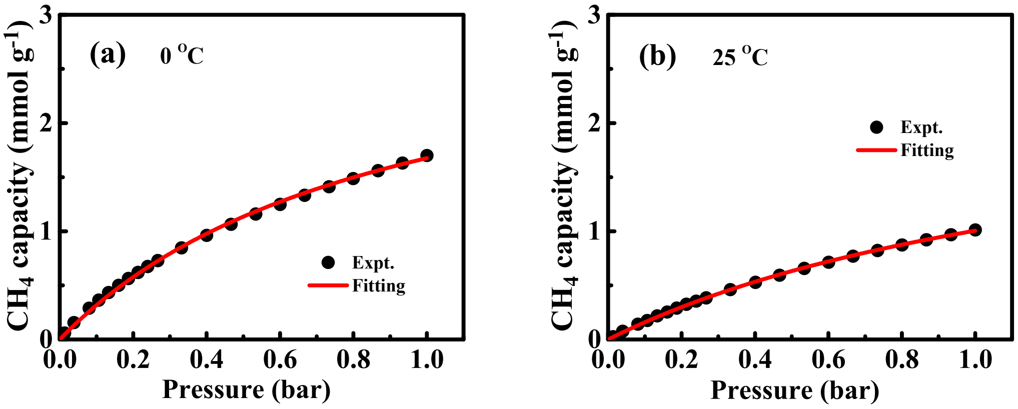

Figure S5. CH_4 gas adsorption for N-WAPC at 0 ºC (a) and 25 ºC (b). The continuous solid line corresponds to the L fittings of the experimental data.

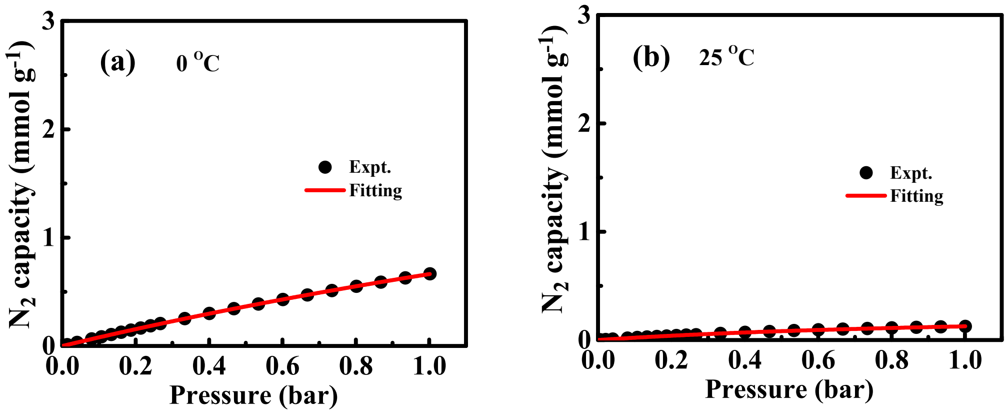

Figure S6. N_2 gas adsorption for N-WAPC at 0 ºC (a) and 25 ºC (b). The continuous solid line corresponds to the L fittings of the experimental data.

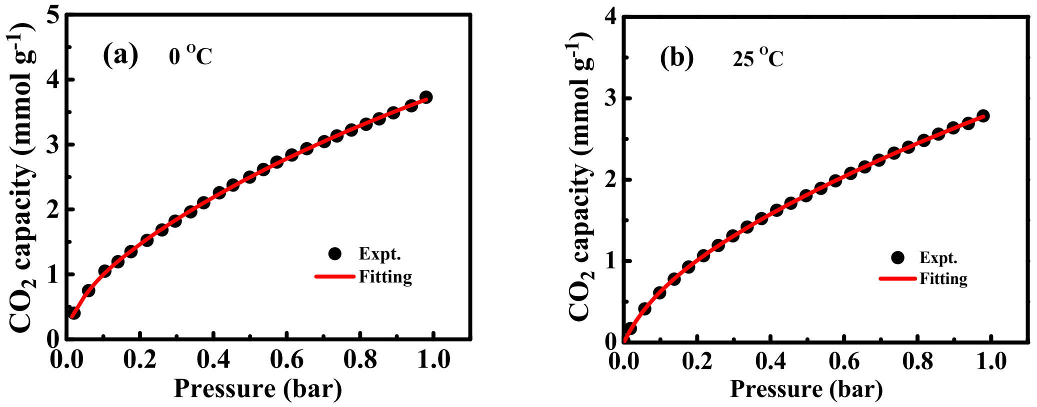

Figure S7. CO_2 gas adsorption for WAPC at 0 ºC (a) and 25 ºC (b). The continuous solid line corresponds to the DSL fittings of the experimental data.

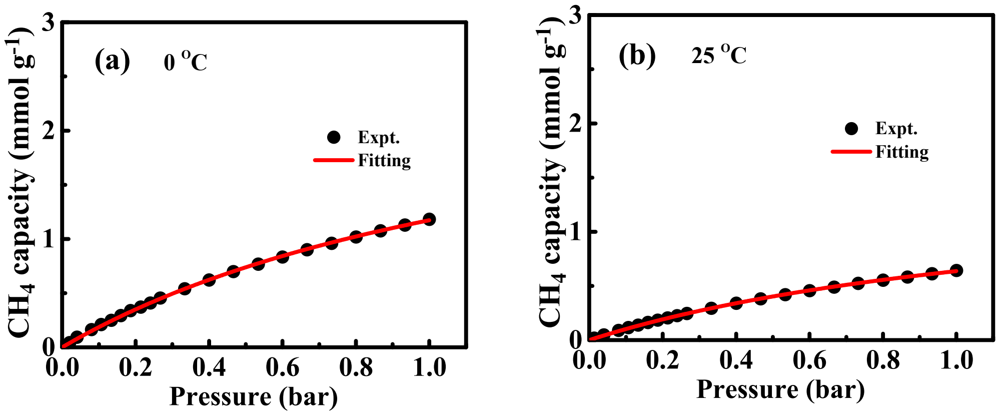

Figure S8. CH_4 gas adsorption for WAPC at 0 ºC (a) and 25 ºC (b). The continuous solid line corresponds to the L fittings of the experimental data.

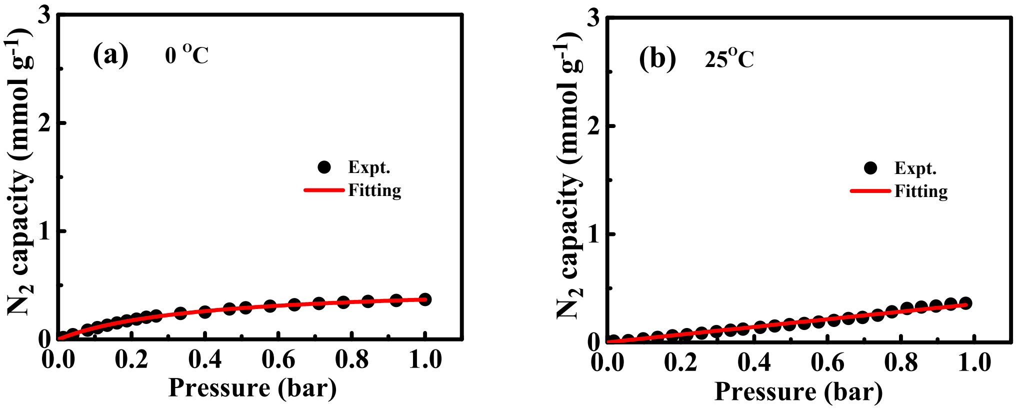

Figure S9. N_2 gas adsorption for WAPC at 0 ºC (a) and 25 ºC (b). The continuous solid line corresponds to the L fittings of the experimental data.

The Langmuir fitting parameters are provided in Table S1, Table S2, and Table S3.

Table S1. Langmuir parameters and coefficient of determination for adsorption of CO_2 in N-WAPC and WAPC.

Samples	Temp.	$q_{sat,A}$	b_A	$q_{sat,B}$	b_B	R^2
N-WAPC	0 °C	6.53734	0.99688	1.26705	29.25162	0.99989
	25 °C	5.96338	0.60985	0.6974	12.71415	0.99998
WAPC	0 °C	9.65753	0.44071	0.81022	26.48572	0.99896
	25 °C	10.74302	0.23779	0.84188	8.03826	0.99994

Table S2. Langmuir parameters and coefficient of determination for adsorption of CH_4 in N-WAPC and WAPC.

Samples	Temp.	$q_{sat,}$	b	R^2
N-WAPC	0 °C	3.19979	1.09927	0.9989
	25 °C	2.45664	0.69263	0.99965
WAPC	0 °C	2.85014	0.6995	0.99971
	25 °C	1.51941	0.72116	0.9997

Table S3. Langmuir parameters and coefficient of determination for adsorption of N_2 in N-WAPC and WAPC.

Samples	Temp.	$q_{sat,}$	b	R^2
N-WAPC	0 °C	3.65378	0.22187	0.99999
	25 °C	0.27746	0.83827	0.99966
WAPC	0 °C	0.50613	2.65687	0.99926
	25 °C	14.41001	0.02517	0.98557
